# The Complete Mitochondrial Genome of *Evergestis extimalis* (Scopoli, 1763) and Its Phylogenetic Implications

**DOI:** 10.3390/biology15151326

**Published:** 2026-08-06

**Authors:** Yunxiang Liu, Yuan Zhao, Yuwei Fan, Qing Song, Yudong Wu, Xiaomei Zhang, Youpeng Lai, Hainan Shao

**Affiliations:** 1 State Key Laboratory of Plateau Ecology and Agriculture, Qinghai Academy of Agriculture and Forestry Sciences, Qinghai University, Xining 810016, China; 2 Xining Botanical Garden, Xining 810008, China; 3 Qinghai Provincial Agricultural Technology Extension Station, Xining 810000, China; 4 Xining Vegetable Technology Service Center, Xining 810016, China

**Keywords:** fennel shoot borer, mitochondrial genome, evolution, phylogeny

## Abstract

The fennel shoot borer, *Evergestis extimalis* (Scopoli) (Lepidoptera: Crambidae: Glaphyriinae), is a widespread and economically significant pest across the Qinghai–Tibet Plateau. However, its geographic distribution, comprehensive genomic resources and the phylogenetic position have not been systematically examined. Our findings reveal a broad distribution for *E. extimalis*, spanning extensive regions across Asia, Europe, and North America. Furthermore, we present the first complete mitochondrial genome (mitogenome) of this species and conduct extensive phylogenetic analyses to clarify its evolutionary relationships within Crambidae. The circular mitogenome is 15,301 bp in length and exhibits typical lepidopteran organization, comprising 13 protein-coding genes (PCGs), 22 transfer RNAs (tRNAs), 2 ribosomal RNAs (rRNAs), and an AT-rich control region. Comparative analysis of *E. junctalis* revealed conserved gene arrangement and high structural similarity. Among the 13 PCGs, *nad6* exhibited the highest nucleotide diversity, whereas *cox1* was the most conserved. Most PCGs were under purifying selection (Ka/Ks < 1) except *atp8*, *nad2*, and *nad6*. The AT-rich region (367 bp) contained tandem repeat motifs characteristic of Pyraloidea. Phylogenetic reconstruction based on concatenated sequences of 13 PCGs and 2 rRNAs from 58 species strongly supported the monophyly of Crambidae and revealed Glaphyriinae as a sister group to Odontiinae. Our results confirm the basal divergence between the “PS clade” (Pyraustinae + Spilomelinae) and the “non-PS clade” (Glaphyriinae, Odontiinae, Crambinae, Scopariinae, Schoenobiinae, and Acentropinae) but reveal discordant relationships within the “CAMMSS clade” (Crambinae, Acentropinae, Midilinae, Musotiminae, Schoenobiinae, and Scopariinae) compared to earlier nuclear gene-based studies. This study fills a significant gap in Glaphyriinae mitogenomics and provides valuable genomic resources for future population genetics on this economically important pest.

## Simple Summary

The fennel shoot borer (*Evergestis extimalis*) is an economically significant pest of rapeseed on the Qinghai–Tibet Plateau; however, its genomic resources and evolutionary relationships have remained largely unexplored. Here, we present the first complete mitochondrial genome of this species, which is 15,301 base pairs in length and exhibits a typical lepidopteran structure with conserved gene content and arrangement. Our phylogenetic analyses place *E. extimalis* within the subfamily Glaphyriinae and clarify its relationships within the family Crambidae. This study provides valuable genomic resources for future research on this pest and contributes to a better understanding of crambid evolution.

## 1. Introduction

Pyraloidea represents one of the most diverse superfamilies within Lepidoptera, encompassing over 16,000 described species distributed globally [1,2]. Within this group, the family Crambidae accounts for approximately 60% of species diversity and includes 15 subfamilies with distinct morphological and ecological traits [1,3]. Glaphyriinae is a relatively small but economically important subfamily of Crambidae, encompassing 137 species across 10 genera. Many glaphyriine species are serious agricultural pests whose larvae mine leaves and flowers or bore into fruits, causing substantial damage to cruciferous crops such as *Brassica napus* L. and cabbage [4,5].

Recent phylogenetic studies have significantly advanced our understanding of subfamily relationships within Pyraloidea. Multilocus nuclear gene analyses by Regier et al. [6] and Léger et al. [1] established a robust phylogenetic framework, identifying two major clades: the “PS clade” (Pyraustinae + Spilomelinae) and the “non-PS clade”, which includes six other subfamilies. However, mitochondrial genome-based studies have revealed conflicting topologies, particularly regarding the placement of Odontiinae and related subfamilies [7]. Critically, no comprehensive mitogenome-based phylogenetic study has yet included Glaphyriinae, leaving a key gap in our understanding of pyraloid evolution. The growing availability of mitogenomic data now makes it possible to conduct comprehensive phylogenomic analyses to test these competing hypotheses [8,9].

*Evergestis extimalis* (Scopoli, 1763) (Lepidoptera: Crambidae: Glaphyriinae), commonly known as the fennel shoot borer, is a univoltine pest widely distributed across Asia and Europe. On the Qinghai–Tibet Plateau, it has emerged as a devastating pest of rapeseed (*Brassica napus* L.), where larval feeding on pods and seeds [10]. Given that this pest completes one generation per year, its damage is concentrated within a critical period, leading to seed loss and yield reductions of up to 50%. These characteristics have made it a growing economic concern in high-altitude agricultural regions [4,11].

Mitochondrial genomes have proven invaluable for phylogenetic inference, species identification, and population genetics due to their maternal inheritance, high copy number, rapid evolution, and general lack of recombination [12,13]. Improved assembly algorithms and annotation tools have made complete mitogenome sequencing routine [14]. In Pyraloidea, mitogenomic data have successfully resolved deep-level phylogenetic relationships and provided insights into speciation processes [15,16]. Beyond their utility in molecular systematics, mitochondrial DNA content and gene expression are finely tuned to meet organ-specific energy demands, underscoring the functional relevance of mitogenomic variation in metabolic adaptation across different tissues and physiological states [17]. Such data also hold promise for tracing population migration patterns and understanding the evolution of insecticide resistance, offering potential insights for pest management [18].

To address the existing knowledge gaps, this study aimed to (1) sequence and characterize the complete mitogenome of *E. extimalis*; (2) conduct comprehensive comparative analyses of mitogenomic features with closely related species; (3) perform robust phylogenetic reconstruction using multiple datasets to resolve the position of Glaphyriinae within Crambidae; (4) compile global distribution records to support future population genetic and phylogeographic research; and (5) evaluate the utility of mitogenomic data for inferring subfamily-level relationships in Pyraloidea. Our findings provide the first mitogenomic perspective on Glaphyriinae evolution and deliver essential genomic resources for future molecular studies of this economically important pest group.

## 2. Materials and Methods

### 2.1. Sample Collection and DNA Extraction

General surveys and targeted site assessments of *E. extimalis* were conducted on the Qinghai–Tibet Plateau (26° N–39° N, 73° E–104° E), China from 2022 to 2024. Larvae were collected from rapeseed fields and identified using molecular technology. For DNA extraction, ten final-instar larvae from each sampling site were processed using the Biospin insect genomic DNA extraction kit (Bioer Technology Co., Ltd., Hangzhou, China) following manufacturer’s protocols. Distribution records were compiled from field surveys and the Global Biodiversity Information Facility (GBIF) Occurrence Download. Available online: https://doi.org/10.48580/dgy8b (accessed on 21 March 2023) [19,20,21]. The distribution map was produced with ArcGlobe 10.8 and finalized using Adobe Photoshop CC 2019. Adult specimens of *E. extimalis* were collected in Xining City, Qinghai Province, China (101°49′17″ E, 36°34′3″ N) between June and July 2023. Fresh specimens were preserved immediately in 95% ethanol and stored at −80 °C for DNA extraction. Species identification was based on morphological characteristics and confirmed by molecular technology.

### 2.2. Mitogenome Sequencing and Assembly

Total genomic DNA was sequenced using next-generation sequencing technology (Illumina HiSeq X10, Biomker Technologies Corporation, Beijing, China). Genomic libraries with an insert size of 350 bp were constructed and sequenced to generate 2 × 150 bp paired-end reads. Raw reads were quality-filtered with Trimmomatic v.0.39 (Usadel Lab., Aachen, Germany) [22] using parameters: LEADING:3, TRAILING:3, SLIDINGWINDOW:4:15, MINLEN:36. Low-quality reads (Phred score < 20) and adapter sequences were removed prior to assembly. Clean reads were assembled into a complete circular mitogenome using NOVOPlasty v.4.3.1 (Interuniversity Institute of Bioinformatics in Brussels, Université Libre de Bruxelles and Vrije Universiteit Brussel, Brussels, Belgiu) [23], with the *cox1* gene of *Evergestis junctalis* (GenBank: NC_030509) as the seed sequence. The assembled mitogenome was deposited in GenBank under accession number OM236669.

### 2.3. Mitogenome Annotation and Comparative Analyses

The mitogenome sequence was annotated with Geneious Prime v.2022.0.1 (Biomatters, Auckland, New Zealand). Transfer RNA (tRNA) genes were identified and positioned using the MITOS Web Server (Bioinformatics, Universität Leipzig, Leipzig, Germany) [24] and compared with homologous mitogenome sequences. Secondary structures of tRNAs, following the anticipated cloverleaf secondary structure, were illustrated using Adobe Illustrator CC2017 based on MITOS outcomes and manual predictions. Protein-coding genes (PCGs) and ribosomal RNA (rRNA) genes were identified by alignment with other Pyraloidea mitogenome sequences. The circular map of the mitogenome was generated with OrganellarGenomeDRAW (OGDRAW) v.131 (Max-Planck-Institut für Molekulare Pflanzenphysiologie, Potsdam-Golm, Germany) [25]. PhyloSuite v.1.2.2 (Lanzhou University, Lanzhou, China) [26] was employed to compute nucleotide composition and generate Relative Synonymous Codon Usage (RSCU) charts. Nucleotide diversity (Pi) analyses and sliding window analyses (window size 200 bp, step size 20 bp) for the 13 PCGs and two rRNA genes were performed using DnaSP v.6.0 (Universitat de Barcelona, Barcelona, Spain) [27]. The rates of nonsynonymous substitutions (Ka), synonymous substitutions (Ks), and their ratio (Ka/Ks) were calculated with DnaSP v.6.0 (Universitat de Barcelona, Barcelona, Spain) [27]. Tandem repeats in the control region were identified using the Tandem Repeats Finder server (Program in Bioinformatics, Boston University, Boston, MA, USA) [28].

### 2.4. Phylogenetic Analysis

Phylogenetic analysis included 58 mitogenome sequences (one newly sequenced, 57 retrieved from GenBank; Table S1). The ingroup comprised 56 Crambidae species, with two Pyralidae species (*Endotricha consocia* Butler and *Hypsopygia regina* Butler) as outgroups. Nucleotide sequences of the 13 PCGs (stop codons excluded) were aligned with MAFFT v.7.490 (Center for Computational Sciences (CBRC), University of the Ryukyus, Okinawa, Japan) [29] using the L-INS-i algorithm and codon alignment mode (genetic code table: invertebrate mitochondrial). The rRNA genes were aligned using MAFFT v.7 (IFReC, Tokyo, Japan) with the G-INS-i algorithm, accounting for secondary structure. Ambiguously aligned regions were removed using Gblocks v.0.91b (Institut de Biologia Evolutiva (IBE), CSIC-UPF, Barcelona, Spain) [30] under relaxed settings. Alignments for individual genes were concatenated using PhyloSuite v.1.2.2 (Institute of Hydrobiology, Chinese Academy of Sciences, Wuhan, China) [26].

The optimal partitioning schemes and substitution models were determined using PartitionFinder v.2.1.1 (Research School of Biology, Australian National University, Canberra, Australia) [31] under the Bayesian Information Criterion (BIC) with the greedy search algorithm. Maximum likelihood (ML) analyses were performed using IQ-TREE v.2.2.0 (The Australian National Univers, Canberra, Australia) [32] with 5000 ultrafast bootstrap (UFB) 5000 replicates and 5000 SH-aLRT replicates. Nodes with UFB ≥ 95 and SH-aLRT ≥ 80 were considered strongly supported. The optimal partitioning scheme and substitution models are presented in (Tables S2–S5). Bayesian inference (BI) analyses were performed using MrBayes v.3.2.7a (Department of Biodiversity Informatics, Swedish Museum of Natural History, Stockholm, Sweden) [33] on the CIPRES Science Gateway. Four Markov chain Monte Carlo (MCMC) chains (one cold, three heated; temperature = 0.2) were run for 10 million generations, sampling every 1000 generations. Convergence was assessed by: (1) ensuring effective sample sizes (ESS) > 200 in Tracer v.1.7.2 (Institute of Evolutionary Biology, University of Edinburgh, Edinburgh, UK); (2) confirming an average standard deviation of split frequencies < 0.01; and (3) verifying that the potential scale reduction factor (PSRF) approached 1.0. The first 25% of sampled trees were discarded as burn-in (Tables S2–S5). Nodes with posterior probability (PP) ≥ 0.95 were considered strongly supported. Phylogenetic trees were visualized using FigTree v.1.4.4 (Institute of Evolutionary Biology, University of Edinburgh, Edinburgh, UK).

## 3. Results and Discussion

### 3.1. Geographic Distribution Patterns

**Figure 1:**
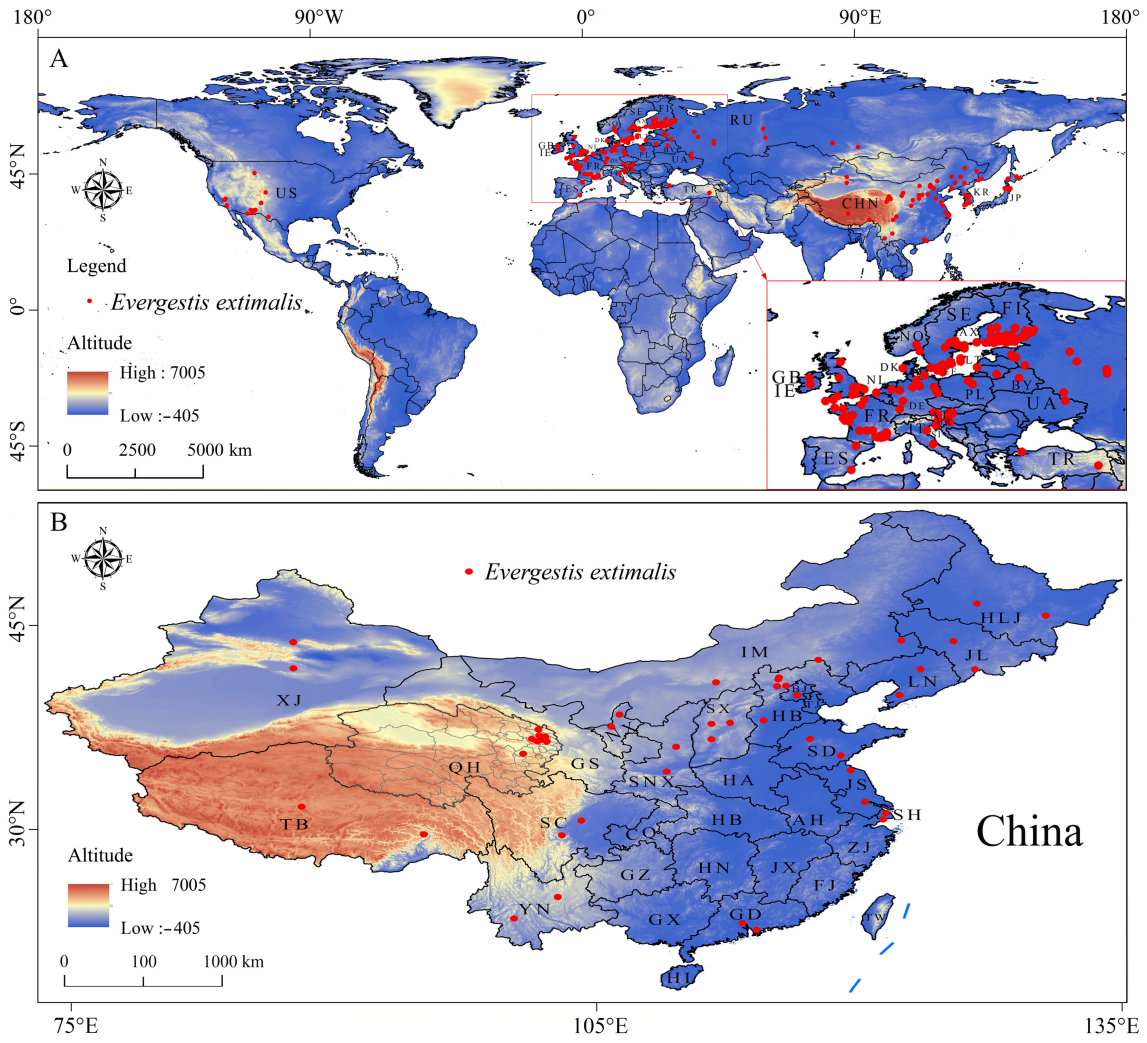
Distribution of *Evergestis extimalis*. (**A**) Global distribution records compiled from GBIF database and field surveys; (**B**) Distribution across China showing 18 provinces with confirmed occurrences.

**Figure 2:**
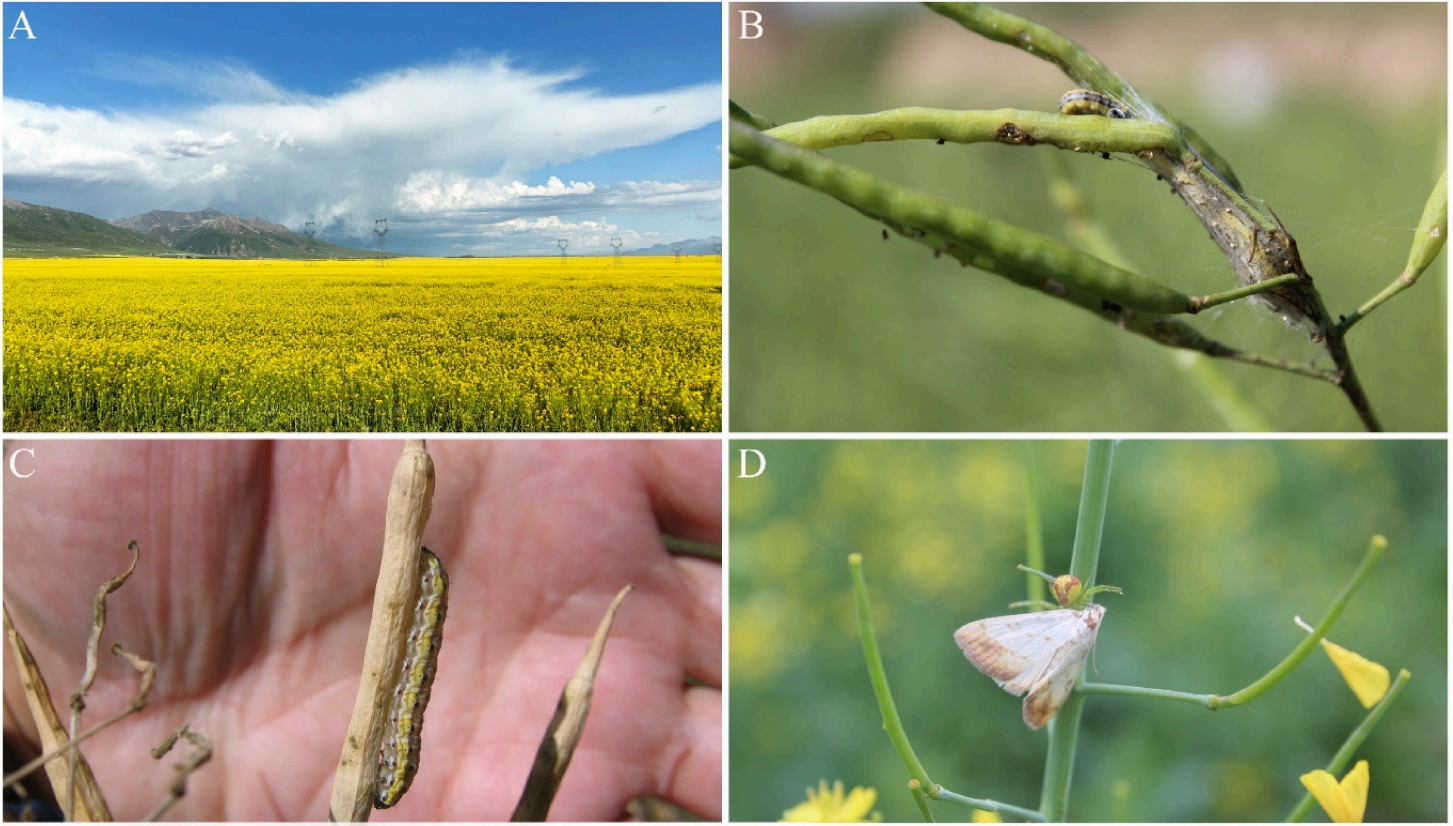
Developmental stages of *E. extimalis*. (**A**) Habitat; (**B**) Pupa; (**C**) Final instar larva; (**D**) Adult specimen.

Combined field surveys and literature reviews indicate a broad distribution for *E. extimalis*, across Asia, Europe, and North America (Figure 1, Table S6). In China, this species has been recorded in 18 provinces, with the highest abundances concentrated in the eastern Qinghai–Tibet Plateau region (Qinghai and Tibet) and in northern provinces (Beijing, Hebei, Heilongjiang). Its altitudinal range extends from lowlands up to 3200 m a.s.l. on the Qinghai–Tibet Plateau, demonstrating remarkable ecological adaptability. In Europe, *E. extimalis* is widely distributed across temperate regions, with confirmed records from the United Kingdom, Ireland, France, Germany, Austria, Slovenia, and Russia. The species shows a distinct preference for agricultural landscapes and appears to have expanded its range alongside the intensification of rapeseed cultivation (Figure 2). *E. extimalis* primarily attacks Brassicaceae species, with *Brassica napus* (rapeseed), *B. oleracea* (cabbage), and *Raphanus sativus* (radish) as the most commonly reported hosts [4,10]. This host specificity provides opportunities for targeted pest monitoring and management.

### 3.2. Mitogenome Organization and Structural Features

**Figure 3:**
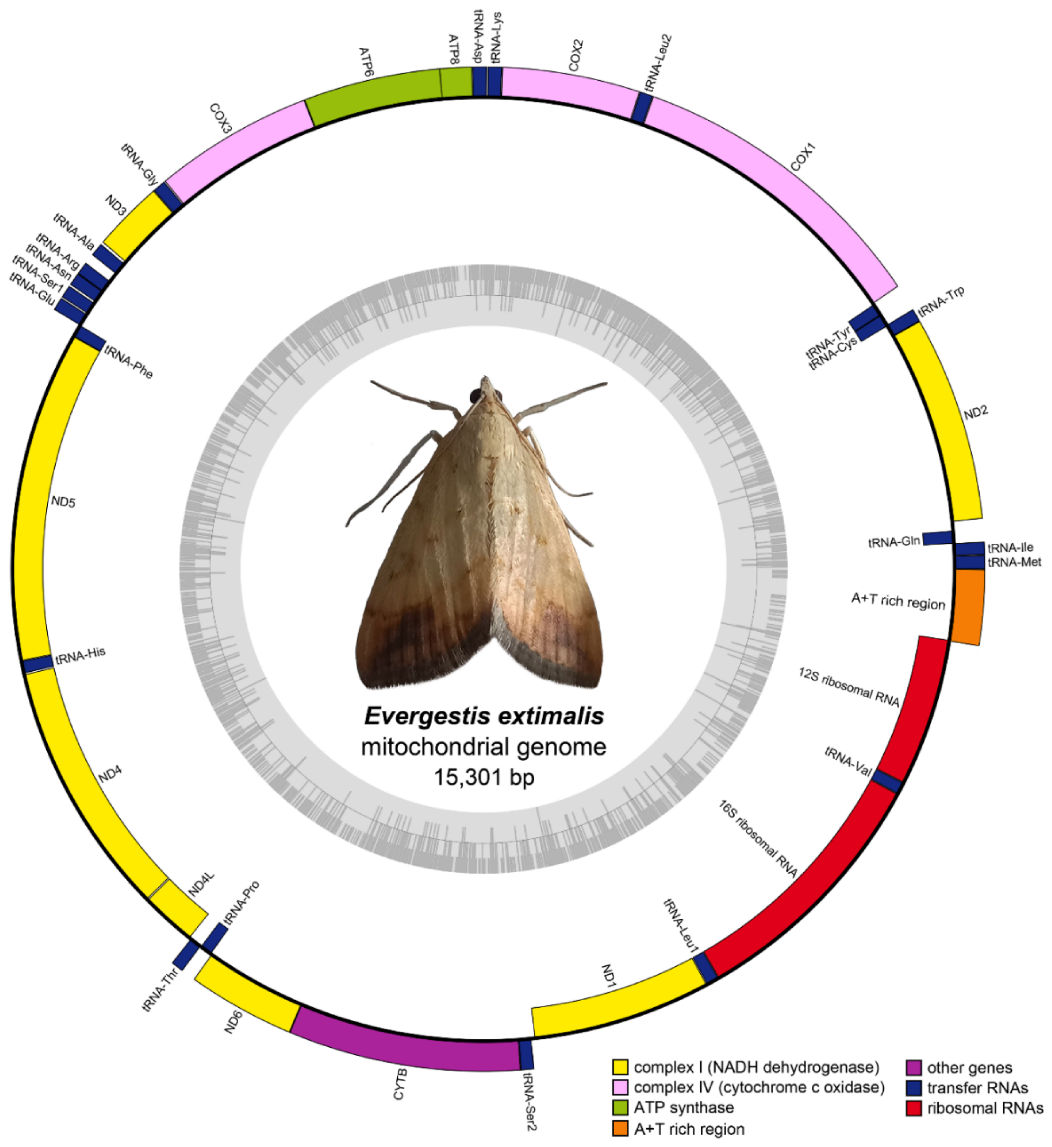
Circular representation of the *E. extimalis* mitochondrial genome. The circular map depicts gene organization with genes on the H-strand (outer circle) and L-strand (inner circle).

The complete mitochondrial genome of *E. extimalis* is a circular, double-stranded DNA molecule of 15,301 bp (GenBank: OM236669). This size falls within the typical range for Crambidae (15,084–15,438 bp) and is similar to that of the closely related *E. junctalis* (15,438 bp) (Table S7). The mitogenome exhibits the canonical lepidopteran gene content of 37 genes: 13 PCGs, 22 tRNAs, two rRNAs, and an AT-rich control region (Table 1, Figure 3).

The gene arrangement follows the ancestral lepidopteran pattern, including the characteristic trnM-trnI-trnQ cluster. Among the 37 genes, 23 are encoded on the majority (H) strand, while the remaining 14 genes (four PCGs: nad5, nad4, nad4L, nad1; eight tRNAs; two rRNAs) are located on the minority (L) strand (Figure 3). This strand bias is consistent across all sequenced pyraloid mitogenomes. The overall nucleotide composition was highly A + T-biased (80.7%), which is lower than other species such as *E. junctalis* Warren (Crambidae, Glaphyriinae), *Conogethes punctiferalis* Guenée (Crambidae, Spilomelinae), *Chilo auricilius* Dudgeo (Crambidae, Crambinae), *Prophantis octoguttalis* Felder & Rogenhofer (Crambidae, Spilomelinae), *Paracymoriza prodigalis* (Leech) (Crambidae, Acentropinae), *Eumorphobotys obscuralis* (Caradja) (Crambidae, Pyraustinae) and *Pyrausta despicata* Scopoli (Crambidae, Pyraustinae) (80.97–82.45%); however, it is slightly higher in the case of *Dausara latiterminalis* Yoshiyasu (Crambidae, Odontiinae), *Scirpophaga incertulas* Walker (Crambidae, Schoenobiinae), *Nyctegretis seminigra* Yang, Zhou, and Ren (Pyralidae, Phycitinae) and *Elophila interruptalis* Pryer (Crambidae, Acentropinae) (77.05–80.60%) [5,7,34,35,36,37]. Nucleotide skew analysis revealed a slight negative AT-skew (−0.018) and a more pronounced negative GC-skew (−0.168) (Table 2), consistent with patterns observed in other crambid species, with the exception of *P. octoguttalis* (0.006), *S. incertulas* (0.031), *P. prodigalis* (0.002), *E. obscuralis* (0.011) and *P. despicata* (0.009). The GC-skewness values were negative across all mitochondrial genomes of Pyraloidea species, indicating a higher proportion of C nucleotides than G nucleotides (Table S8).

The overall nucleotide composition was highly A + T-biased (80.7%), which is lower than other species such as *E. junctalis*, *C. punctiferalis*, *C. auricilius*, *P. octoguttalis*, *P. prodigalis*, *E. obscuralis* and *P. despicata* (80.97–82.45%); however, it is slightly higher in the case of *D. latiterminalis*, *S. incertulas*, *N. seminigra* and *E. interruptalis* (77.05–80.60%) [5,7,37,38,39,40]. Nucleotide skew analysis revealed a slight negative AT-skew (−0.018) and a more pronounced negative GC-skew (−0.168) (Table 2), consistent with patterns observed in other crambid species, with the exception of *P. octoguttalis* (0.006), *S. incertulas* (0.031), *P. prodigalis* (0.002), *E. obscuralis* (0.011) and *P. despicata* (0.009). The GC-skewness values were negative across all mitochondrial genomes of Pyraloidea species, indicating a higher proportion of C nucleotides than G nucleotides (Table S8).

The mitogenome contains 11 overlapping intergenic regions (1–8 bp) and 14 intergenic spacers totaling 156 bp. The longest intergenic spacer (48 bp) occurred between trnR and trnN. Notably, a conserved 7 bp overlap between atp8 and atp6 (ATGATAA) was present, a hallmark feature of all Pyraloidea mitogenomes sequenced to date [15,38].

### 3.3. Base Composition and Codon Usage

The AT content varied across different genomic regions: PCGs (79.2%), tRNAs (81.0%), rRNAs (85.4%), and the AT-rich region (95.1%) (Table 2). This compositional heterogeneity is typical of insect mitogenomes and reflects different functional constraints [12,39]. The exceptionally high AT content in the control region is thought to facilitate DNA unwinding during replication initiation.

**Figure 4:**
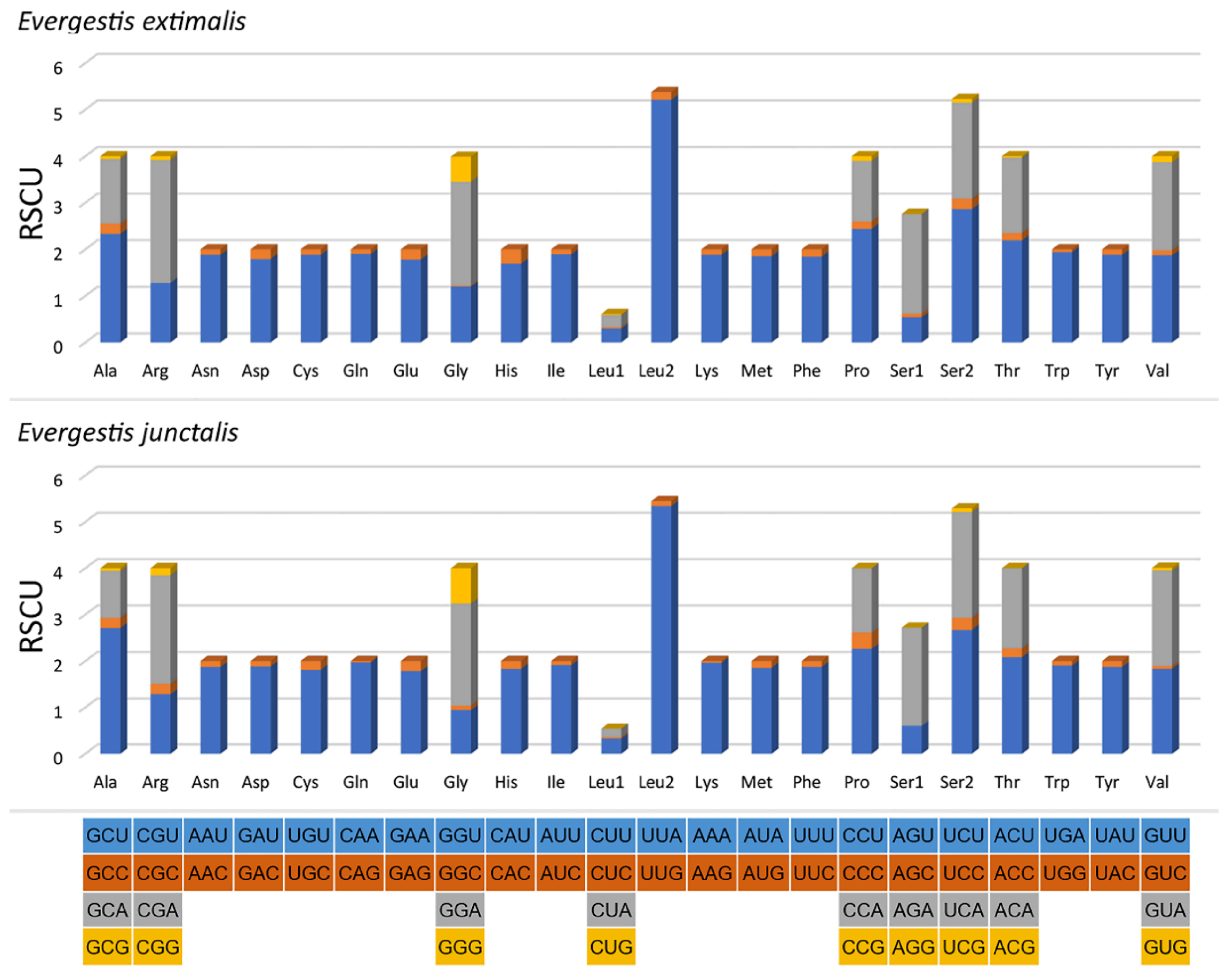
Relative Synonymous Codon Usage (RSCU) in two Pyraloidea moths. The bar graph illustrates the relative synonymous codon usage across all amino acid codons. Bars are color-coded according to the codon labels. Codon families are indicated below the X axis.

Codon usage analysis revealed that UUA (Leu2), AUU (Ile), UUU (Phe), and AUA (Met) were the most frequently used codons in both *E. extimalis* and *E. junctalis* (Figure 4). The usage frequency widely observed in Pyralidae mitogenomes, such as *Chilo infuscatellus* Snellen, *Cydalima perspectalis* (Walker), *E. consocia*, *H. regina*, *Orybina plangonalis* Walker, *Tyspanodes striata* (Butler), and *Maruca vitrata* (Fabricius) [15,34,40]. Leu2, Ile, and Phe codon families showed the highest usage rates, with isoleucine showing the highest RSCU value. This codon usage bias is strongly correlated with the A + T content bias and is consistent with patterns observed in other moths [41,42].

### 3.4. Protein-Coding Genes and Evolutionary Rates

**Figure 5:**
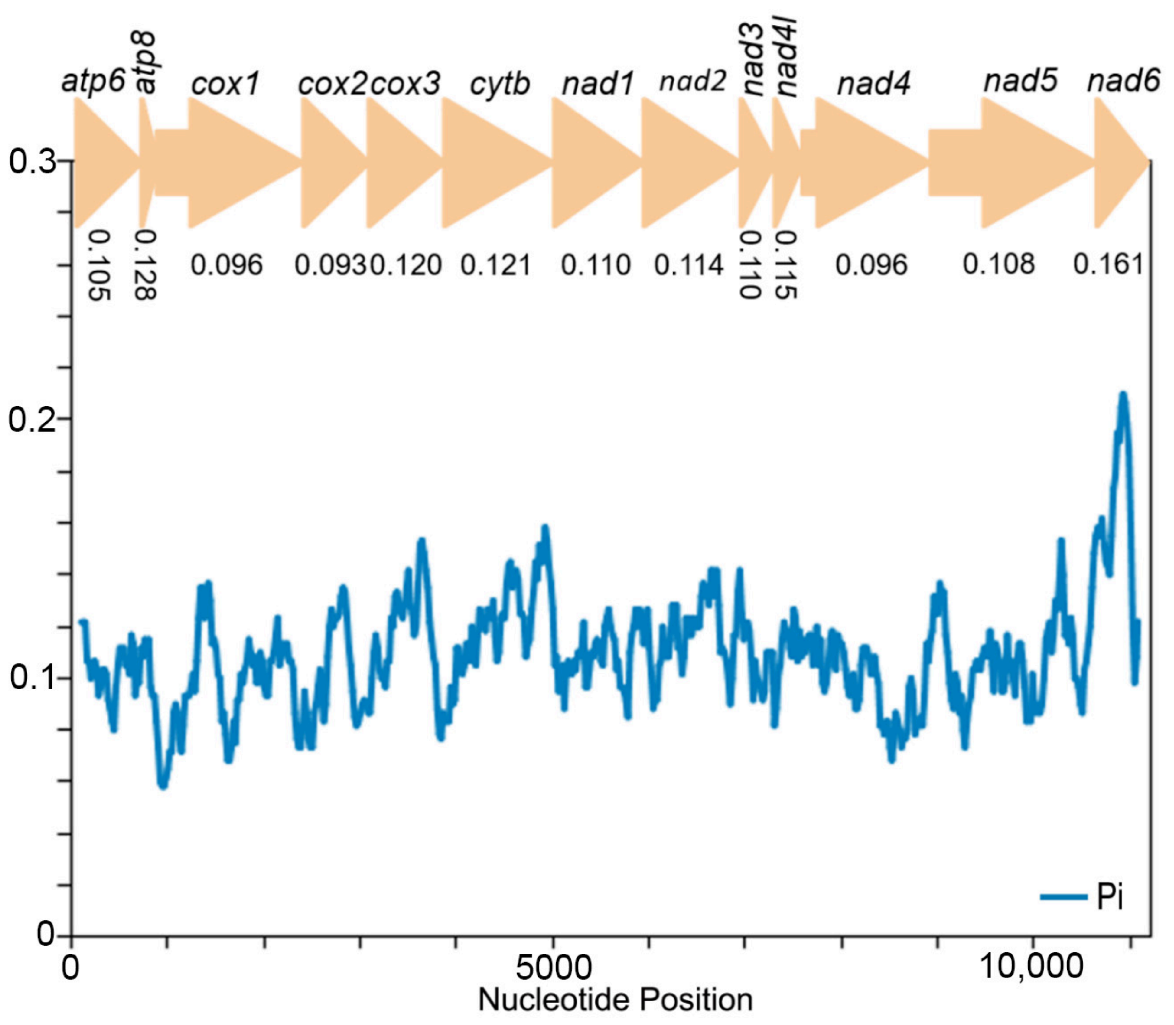
Sliding window analysis of the 13 PCGs and two rRNAs. Nucleotide diversity (Pi) values calculated with window size 200 bp and step size 20 bp.

**Figure 6:**
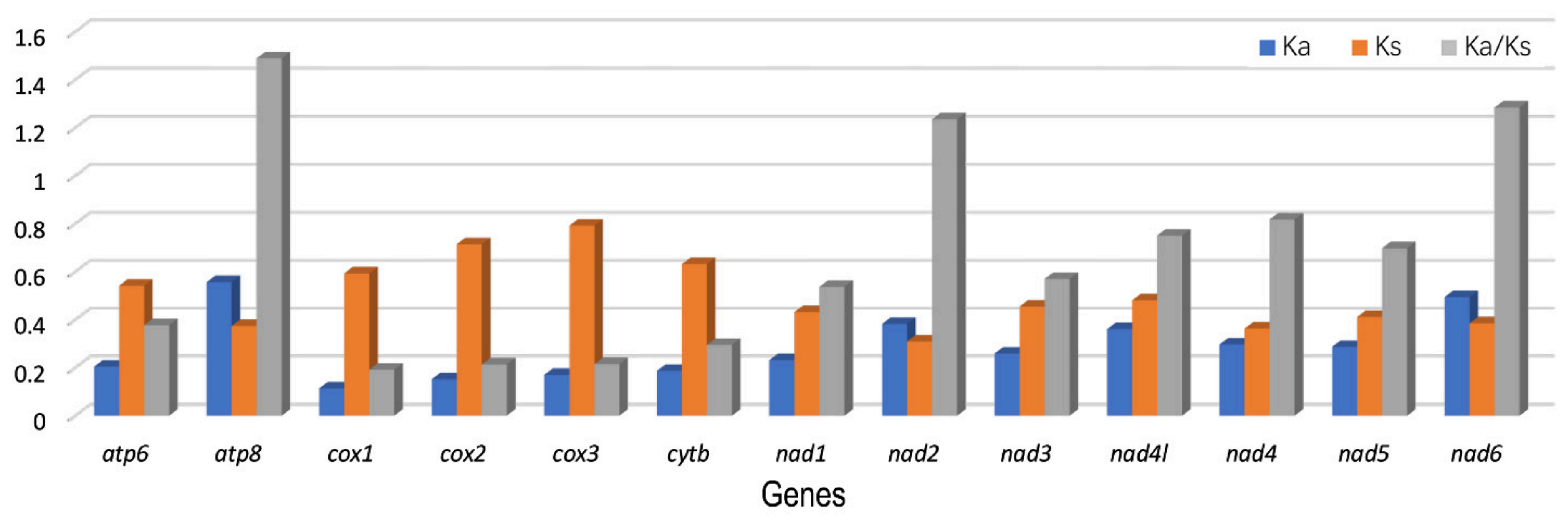
Evolutionary rates of the mitochondrial genomes of two Pyralidae moths. Ka (nonsynonymous), Ks (synonymous), and Ka/Ks ratios of the PCGs in the mitochondrial genomes are calculated with *E. junctalis* as reference.

The total length of PCGs of *E. extimalis* was 11,202 bp, encoding 3733 amino acids. All PCGs initiated with a conventional start codon ATN (ATA/T/G/C), except for *cox1*, which started with CGA—a pattern identical to other pyraloid species [15,43]. Two PCGs (*cox1* and *cox2*) had incomplete stop codons (T nucleotide), while the others terminated with the typical stop codon TAA. This incomplete stop codon, common in lepidopteran insects, is believed to be completed by post-transcriptional polyadenylation [44]. Although termination codons using TAG have been reported in some sequenced Crambidae species—for example, nad6 and nad1 use TAG as their stop codon in *N. seminigra.* mitogenome and *N. triangulella* mitogenome; *nad4l* in *E. obscuralis* and *Demobotys pervulgalis* Hampson is terminated with TAG [36,37]. Sliding-window analysis of nucleotide diversity (Pi) revealed that nad6 was the most variable region, while *cox1* showed the lowest nucleotide diversity (Figure 5). The conserved nature of *cox1* supports its widespread use as a DNA barcoding marker [45]. The Ka/Ks ratios for most PCGs were below 1, indicating purifying selection (Figure 6). However, *atp8*, *nad2*, and *nad6* showed Ka/Ks ratios > 1, suggesting relaxed selection or potential adaptive evolution [46]. These genes may therefore be subject to different selective pressures compared to other mitochondrial genes.

### 3.5. Transfer RNA Genes, Ribosomal RNA Genes, and AT-Rich Region

**Figure 7:**
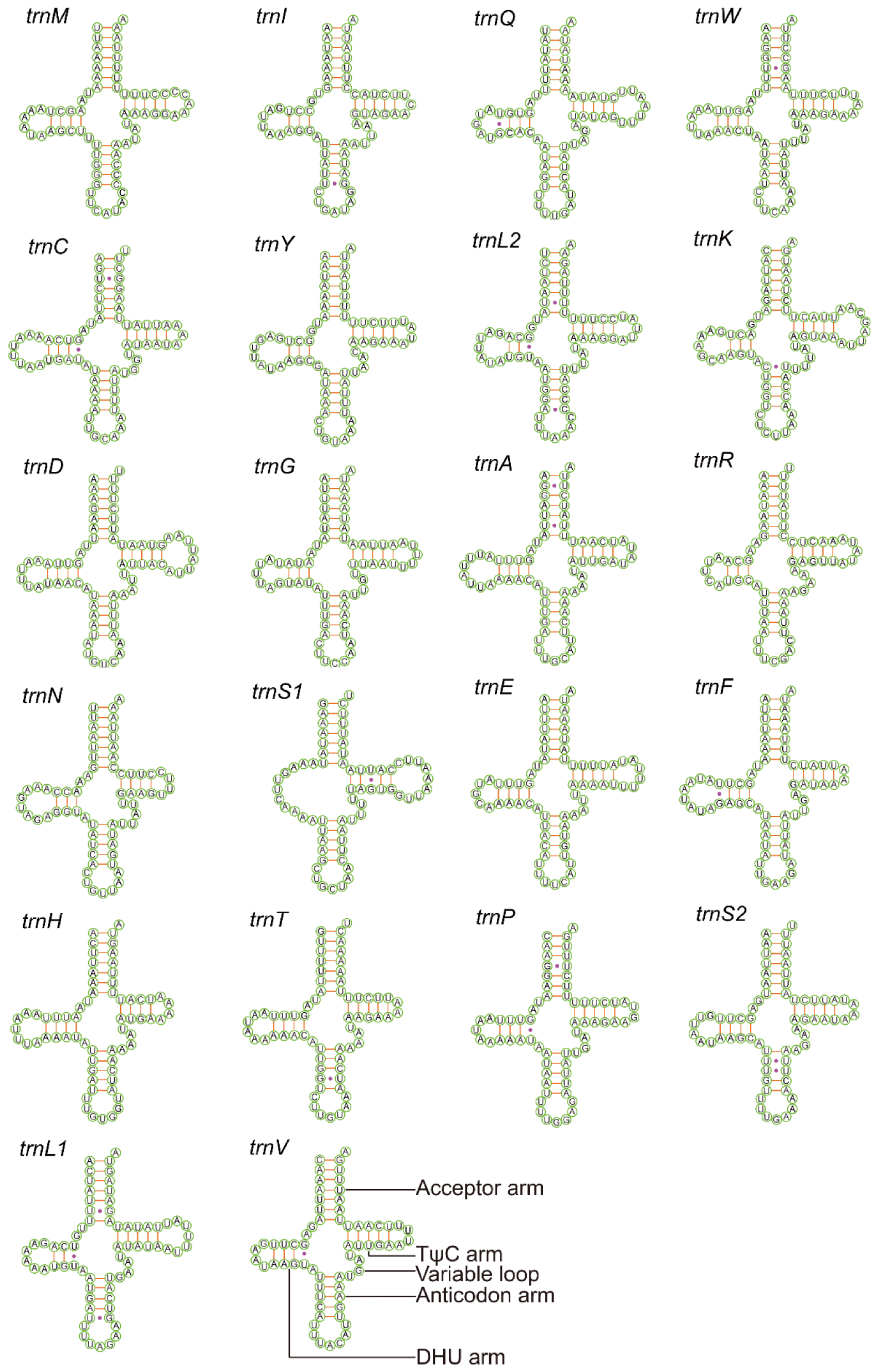
Secondary structures of 22 tRNAs within the *E. extimalis* mitogenome. Lines (-) depict Watson–Crick base pairs, dots (·) represent mismatched pairs.

The mitogenome encodes 22 tRNA genes, ranging from 65 to 71 bp in length. Among these, eight tRNAs are encoded on the L-strand and fourteen on the H-strand (Table 1). Except for *trnS1* (AGN), all tRNAs are predicted to fold into the typical cloverleaf secondary structure; *trnS1* lacks the dihydrouridine (DHU) arm (Figure 7), a feature commonly observed in many insect taxa [47]. A total of 22 unmatched base pairs were identified within the tRNA repertoire, comprising 15 non-canonical G-U pairs and seven mismatches (five U-U, one A-C, and one C-U). These mismatches likely result from ancient nucleotide substitutions or post-transcriptional insertional editing events [48]. The two ribosomal RNA genes, *rrnL* (1344 bp) and *rrnS* (778 bp), were identified with conserved structural organization and genomic locations. Their AT contents are 84.4% and 85.6%, respectively (Table 2), consistent with those reported for other pyraloid mitogenomes [7]. Beyond their canonical roles in translation, the 22 tRNA genes identified in this genome may have broader regulatory functions. Accumulating evidence from diverse taxa, including mammals, demonstrates that mitochondrial-encoded non-coding RNAs serve as critical modulators of cellular energetics and metabolic adaptation, suggesting that their functional significance extends well beyond structural scaffolding [49].

**Figure 8:**
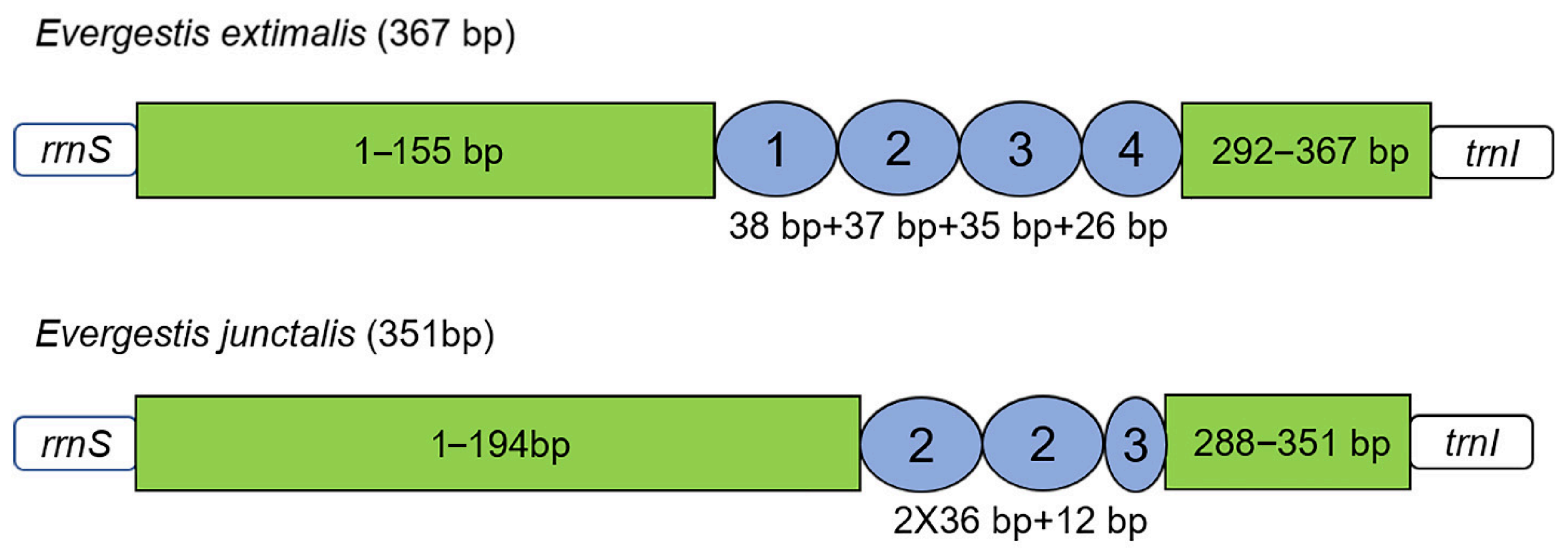
Architectural layout of AT-rich regions of *E. extimalis* and *E. junctalis*. Blue ellipses indicate tandem repeat units; green boxes represent regions without repeats.

**Figure 9:**
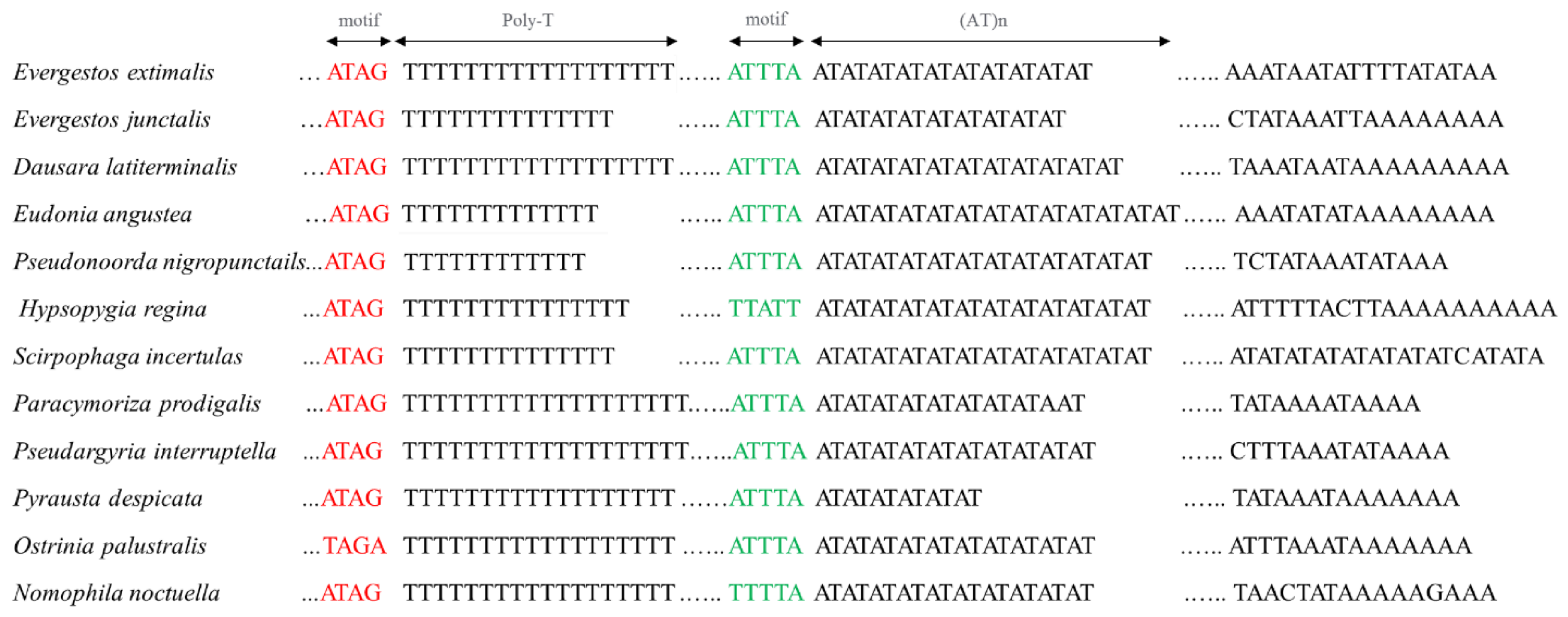
The A + T-rich regions of the typical subfamilies of Cramibidae. The conserved motifs ATAG and ATTTA are marked as red and green, respectively. Dots represent omitted sequences.

The AT-rich regions of *E. extimalis* and *E. junctalis* were 367 bp and 351 bp long, respectively, located between *rrnS* and *trnM* genes (Figure 8). These lengths are similar to those in other mitogenomes of Pyraloidae (about 340 bp on average). These regions are involved in the regulation of transcription and replication. Tandem repeat analysis revealed that *E. extimalis* contains an array of absolute tandem repeats located within the AT-rich region (positions 156–291). Similarly, *E. junctalis* contains repeats between positions 195 and 287 (Figure 8). Tandem repeats are believed to play significant roles in DNA methylation, gene transcription, and replication control [50,51]. The comparison of the control regions from representatives of 10 subfamilies of Cramibidae is shown in Figure 9. Different lengths of the A + T-rich regions are observed across various subfamilies; however, several conserved elements were also identified, including the motif ATAG, the subsequent large poly-T stretch, the motif ATTTA, microsatellite structures (AT)n, and finally, the poly-A stretch (Figure 9). These conserved blocks are considered to play a key role in regulating the replication and transcription stability across a wide range of eukaryotes [51]. As in other metazoans, the mitochondrial D-loop region—a non-coding segment lacking protein-coding genes—serves as a critical regulatory element governing both replication initiation and transcriptional control. This region exhibits a high propensity for mutations, and nucleotide substitutions within the D-loop can impair transcriptional fidelity and thereby modulate mitochondrial function [52]. In line with this paradigm, the conserved ATAG and ATTTA motifs identified in the control region of *E. extimalis* are likely to exert analogous regulatory influences on mitochondrial replication and transcription dynamics, reinforcing their evolutionary conservation across divergent lineages.

### 3.6. Phylogenetic Relationships and Implications for Crambidae Classification

**Figure 10:**
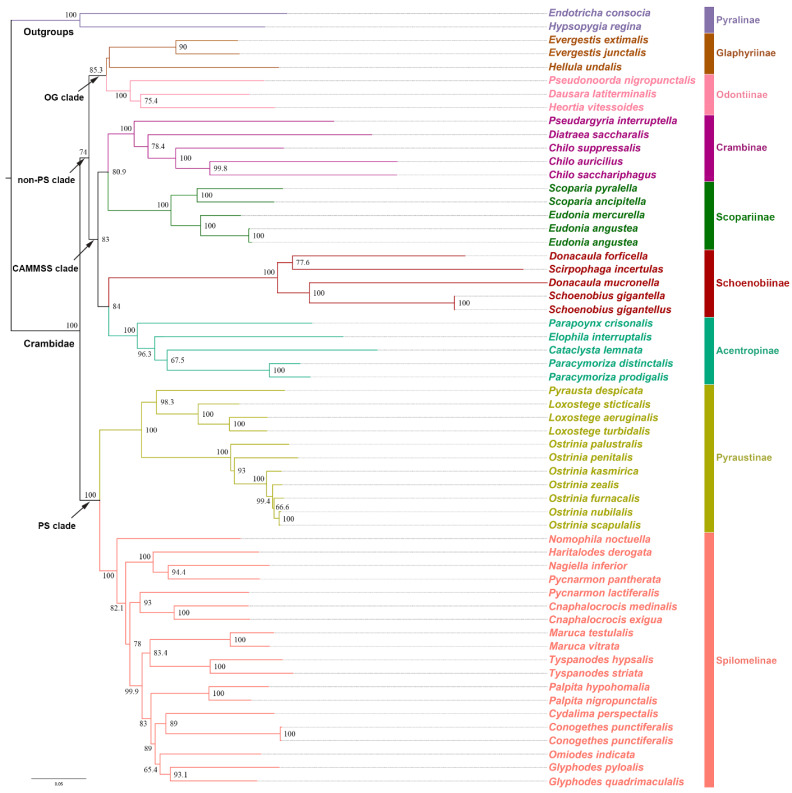
Maximum Likelihood phylogenetic tree of Crambidae based on 13 PCGs and two rRNAs. Numbers at nodes represent bootstrap support values (BS). Scale bar represents substitutions per site. Major clades (“PS clade”, “OG clade”, “CAMMSS clade”) indicated with vertical bars.

**Figure 11:**
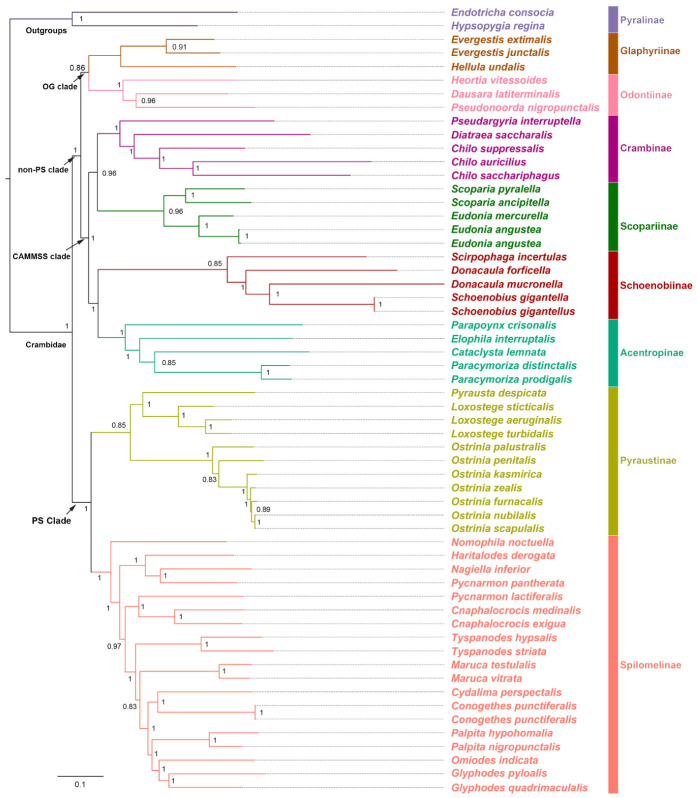
Bayesian Inference phylogenetic tree of Crambidae based on 13 PCGs and two rRNAs. Numbers at nodes represent Bayesian posterior probabilities (PPs).

Phylogenetic analyses based on concatenated nucleotide sequences of 13 PCGs and two rRNAs from 58 pyraloid species yielded highly congruent topologies under both Maximum Likelihood (ML) and Bayesian Inference (BI) methods (Figure 10 and Figure 11). Both analyses strongly supported the monophyly of Crambidae (ML: BS = 100; BI: PP = 1.0) and its sister relationship with Pyralidae (ML: BS = 100; BI: PP = 1.0), consistent with all previous molecular studies [1,6,7,53]. The only topological discrepancy between the maximum likelihood (ML) and Bayesian inference (BI) trees involved the branching order among *Pseudonoorda nigropunctalis* (Hampson), *D. latiterminalis*, and *Heortia vitessoides* (Moore). In the ML topology, the clade containing *P. nigropunctalis* was resolved as sister to a clade comprising *D. latiterminalis* and *H. vitessoides*, whereas the BI topology placed *H. vitessoides* as the earliest diverging lineage, with *P. nigropunctalis* and *D. latiterminalis* forming a sister pair. Aside from this localized incongruence, the two phylogenetic reconstructions were fully concordant with respect to all other terminal relationships. Within Crambidae, two major clades were recovered: (i) the “PS clade” comprising Pyraustinae and Spilomelinae (ML: BS = 100; BI: PP = 1.0), and (ii) the “non-PS clade” containing the remaining six subfamilies (Glaphyriinae, Odontiinae, Crambinae, Scopariinae, Schoenobiinae, and Acentropinae). This deep split is morphologically supported by the absence of anepisternal scale organs in adult Pyraustinae [54].

Novel insights into Glaphyriinae phylogenetic position. This study provides important mitogenome-based evidence supporting the placement of Glaphyriinae within Crambidae. Our analyses robustly place Glaphyriinae as a sister group to Odontiinae, together forming the “OG clade” (ML: BS = 85.3; BI: PP = 0.86) (Figure 10 and Figure 11). This sister-group relationship received moderate-to-strong support across all analyses and is congruent with recent multilocus nuclear gene studies [1]. Within Glaphyriinae, *E. extimalis* and *E. junctalis* constituted a robustly supported monophyletic group (ML: BS = 90%; BI: PP = 0.91), a topological outcome that corroborates their previously recognized morphological affinities. Interestingly, while previous studies reported that *Hellula undalis* also assigned to Glaphyriinae, exhibited a closer phylogenetic affinity to *P. nigropunctalis*—a representative of Odontiinae—than to members of the same subfamily, our phylogenetic analyses recovered Glaphyriinae as a moderately support monophyletic clade in both ML and BI trees (ML: BS = 85.3%; BI: PP = 0.86). This unexpected placement contrasts with earlier mitogenomic findings, that suggested paraphyly of Glaphyriinae based on PCG 12 and PCG 123 [1,7]. This discrepancy warrants further investigationIt may be attributable to the fact that previous phylogenetic studies of Pyraloidea incorporated either one (*E. junctalis*) or two (*E. junctalis*, and *H. undalis*) mitochondrial genomes from Glaphyriinae. By contrast, our expanded dataset, including all three current available mitogenomes for this subfamily, recovered Glaphyriinae as a monophyletic clade. This result provides an alternative phylogenetic hypothesis to the paraphyly of Glaphyriinae proposed in earlier studies [7].

Discordant relationships within the “CAMMSS clade”. Within the “non-PS clade,” our analyses recovered the topology ((Odontiinae + Glaphyriinae) + (Scopariinae + Acentropinae) + (Schoenobiinae + Crambinae)) with variable support (Figure 10 and Figure 11). This mitogenome-based topology is incongruent with previous reconstructions derived from nuclear datasets trees [1,7,55], underscoring a persistent discordance in the inferred subfamilial relationships. Several interrelated factors may account for this topological disparity. First, mitochondrial and nuclear genes exhibit different evolutionary rates and modes of inheritance (maternal versus biparental), which may reflect distinct evolutionary trajectories and thus yield conflicting phylogenetic signals. Second, the “CAMMSS clade” may have undergone rapid diversification events, resulting in short internal branches that constrain phylogenetic informativeness and potentially undermine topological robustness. Third, discrepancies in taxon sampling and substitution model selection could further confound the inferred relationships [56,57,58]. Collectively, these considerations warrant a cautious interpretation of our results. Future efforts integrating additional nuclear loci—ideally through transcriptomic or genomic-scale approaches—will be essential to achieve a more definitive resolution of the interfamilial relationships within the “CAMMSS clade”.

Our results support the monophyly of Crambidae and the major PS/non-PS split but highlight persistent uncertainty in resolving relationships within the “CAMMSS clade.” We recommend: (i) sequencing additional mitogenomes from undersampled subfamilies; (ii) incorporating nuclear genes to generate phylogenomic datasets; (iii) applying coalescent-based species tree methods; and (iv) evaluating alternative partitioning strategies and site-heterogeneous models to reduce systematic error. The placement of Glaphyriinae as sister to Odontiinae suggests these subfamilies may share common ecological traits influencing their diversification, warranting comparative studies of host plant use and resistance mechanisms for integrated pest management.

## 4. Conclusions

This study presents the first complete mitochondrial genome of *E. extimalis* and provides novel phylogenetic perspectives on the evolution of Glaphyriinae. The 15,301 bp mitogenome exhibits typical lepidopteran structure, with conserved gene content and arrangement. Phylogenetic analyses robustly support Glaphyriinae as the sister group to Odontiinae within Crambidae, thereby providing important evidence for the evolutionary status in pyraloid phylogenomics. However, observed topological conflicts with nuclear gene trees underscore the importance of adopting integrative phylogenomic approaches that combine mitochondrial and nuclear markers. The distribution records and genomic resources presented here establish a foundation for future population genetic and phylogeographic research, and will support the development of molecular-based management strategies for this economically important rapeseed pest.

## Figures and Tables

**Table 1:** Organization of the mitochondrial genome in *Evergestis extimalis*.

Gene Name	Location		Size (bp)	Intergenic Nucleotides	Codon		Strand
	From	To			Start	Stop	
*trnM*	1	68	68				H
*trnI*	69	133	65				H
*trnQ*	131	199	69	−3			L
*nad2*	245	1258	1014	45	ATT	TAA	H
*trnW*	1257	1324	68	−2			H
*trnC*	1317	1384	68	−8			L
*trnY*	1384	1450	67	−1			L
*cox1*	1455	2985	1531	4	CGA	T	H
*trnL2*	2986	3052	67				H
*cox2*	3053	3734	682		ATG	T	H
*trnK*	3735	3805	71				H
*trnD*	3809	3879	71	3			H
*atp8*	3880	4041	162		ATC	TAA	H
*atp6*	4035	4715	681	−7	ATG	TAA	H
*cox3*	4715	5503	789	−1	ATG	TAA	H
*trnG*	5506	5572	67	2			H
*nad3*	5573	5926	354		ATT	TAA	H
*trnA*	5937	6005	69	10			H
*trnR*	6054	6119	66	48			H
*trnN*	6119	6184	66	−1			H
*trnS1*	6192	6257	66	7			H
*trnE*	6262	6329	68	4			H
*trnF*	6328	6394	67	−2			L
*nad5*	6394	8127	1734	−1	ATT	TAA	L
*trnH*	8128	8193	66				L
*nad4*	8198	9538	1341	4	ATG	TAA	L
*nad4L*	9542	9835	294	3	ATG	TAA	L
*trnT*	9843	9907	65	7			H
*trnP*	9908	9973	66				L
*nad6*	9976	10,509	534	2	ATT	TAA	H
*cytb*	10,509	11,657	1149	−1	ATG	TAA	H
*trnS2*	11,657	11,723	67	−1			H
*nad1*	11,740	12,678	939	16	ATG	TAA	L
*trnL1*	12,680	12,747	68	1			L
*rrnL*	12,748	14,091	1344				L
*trnV*	14,092	14,156	65				L
*rrnS*	14,157	14,934	778				L
A + T rich region	14,935	15,301	367				H

**Table 2:** Base composition and skewness of mitochondrial genomes of *E. extimalis* and *E. junctalis*.

Feature	Length (bp)	A + T%	AT-Skew	GC-Skew
*E. extimalis*/*E. junctalis*				
Whole genome	15,301/15,438	80.7/81	−0.018/−0.015	−0.168/−0.168
PCGs	11,202/11,205	79.2/79.5	−0.146/−0.141	0.047/0.044
tRNAs	1480/1480	81/81.6	0.013/0.029	0.132/0.158
rRNAs	2122/2123	85.4/85	0.028/0.045	0.331/0.335
A + T rich region	367/351	95.1/94.3	−0.072/−0.069	−0.222/−0.2

## Data Availability

Representative nucleic acid sequences reported in this paper have been submitted to the GenBank (accession number: OK135168OZ).

## Supplementary Materials

The following supporting information can be downloaded at: https://www.mdpi.com/article/10.3390/biology15151326/s1, Table S1. The species used in phylogenetic analyses. Table S2. Optimal partitioning schemes and substitution models for the PCG123 dataset (13 protein-coding genes) of 58 Pyraloidea species used in maximum likelihood (ML) phylogenetic analyses. Table S3. Optimal partitioning schemes and substitution models for PCG123 dataset (13 protein-coding genes) of 58 Pyraloidea species used in Bayesian inference (BI) phylogenetic analyses. Table S4. Optimal partitioning schemes and substitution models for PCG123 + rRNA dataset (13 protein-coding genes + 2 ribosomal RNAs) of 58 Pyraloidea species used in maximum likelihood (ML) phylogenetic analyses. Table S5. Optimal partitioning schemes and substitution models for PCG123 + rRNA dataset (13 protein-coding genes + 2 ribosomal RNAs) of 58 Pyraloidea species used in Bayesian inference (BI) phylogenetic analyses. Table S6. Geographic Distribution of *E. extimalis*. Table S7. List of mitochondrial genomes of Crambidae species for comparison with *E. extimalis*. Table S8. Composition and skewness in the Crambidae mitochondrial genomes. Figure S1. Polymerase chain reaction (PCR) analysis to confirm the larvae of *E. extimalis* by targeting the *COI* (cytochrome c oxidase subunit I) gene.
