# Supplementary material for: The Complete Mitochondrial Genome of Evergestis extimalis (Scopoli, 1763) and Its Phylogenetic Implications
Source: Biology (Basel). 2026 Aug 6;15(15):1326. doi: 10.3390/biology15151326 (PMC13465356; doi:10.3390/biology15151326)
Supplement: Supplementary file 1 [file biology-15-01326-s001.zip › biology-4436450-supplementary.pdf]

SUPPORTING INFORMATION

The Complete Mitochondrial Genome of *Evergestis extimalis* (Scopoli, 1763) (Lepidoptera: Crambidae) and Its Phylogenetic Implications

APPENDIX 1 Additional supporting tables

Table S1 The species used in phylogenetic analyses.

Table S2 Optimal partitioning schemes and substitution models for the PCG123 dataset (13 protein-coding genes) of 58 Pyraloidea species used in maximum likelihood (ML) phylogenetic analyses.

Table S3 Optimal partitioning schemes and substitution models for PCG123 dataset (13 protein-coding genes) of 58 Pyraloidea species used in Bayesian inference (BI) phylogenetic analyses.

Table S4 Optimal partitioning schemes and substitution models for PCG123 + rRNA dataset (13 protein-coding genes + 2 ribosomal RNAs) of 58 Pyraloidea species used in maximum likelihood (ML) phylogenetic analyses.

Table S5 Optimal partitioning schemes and substitution models for PCG123 + rRNA dataset (13 protein-coding genes + 2 ribosomal RNAs) of 58 Pyraloidea species used in Bayesian inference (BI) phylogenetic analyses.

Table S6 Geographic Distribution of *E. extimalis.*

Table S7 List of mitochondrial genomes of Crambidae species for comparison with *E. extimalis*.

Table S8 Composition and skewness in the Crambidae mitochondrial genomes.

Fig S1. Polymerase chain reaction (PCR) analysis to confirm the larvae of *E. extimalis* by targeting the *COI* (cytochrome c oxidase subunit I) gene.

Table S1 The species used in phylogenetic analyses.

Family	Subfamily	Species	Accession number	Reference
Pyralidae	Pyralinae	*Endotricha consocia*	NC_037501	Gschloessl et al., (2020)
		Hypsopygia regina	KP327714	Dai et al., (2018)
Crambidae	Odontiinae	*Heortia vitessoides*	NC_056800	Tang et al., (2023)
		Pseudonoorda nigropunctalis	NC_056801	Unpublished
		Dausara latiterminalis	NC_056799	Unpublished
	Glaphyriinae	*Hellula undalis*	KJ636057	Liu et al., (2021)
		Evergestis junctalis	NC_030509	Gschloessl et al., (2020)
		Evergestis extimalis	OM236669	This study
	Scopariinae	*Scirpophaga incertulas*	NC_031329	Tang et al., (2023)
		Schoenobius gigantella	OZ012565	Unpublished
		Schoenobius gigantellus	OZ392371	Unpublished
		Donacaula mucronella	OZ360547	Unpublished
		Donacaula forficella	OZ377310	Weddle, 2021
	Acentropinae	*Parapoynx crisonalis*	NC_031151	Gschloessl et al., (2020)
		Elophila interruptalis	NC_021756	Park et al., (2019)
		Cataclysta lemnata	NC_050323	Liu et al., (2021)
		Paracymoriza prodigalis	NC_020094	He et al., (2015)
		Paracymoriza distinctalis	NC_023471	Gschloessl et al., (2020)
	Schoenobiinae	*Eudonia angustea*	KJ508052	Yang et al., (2018)
		Eudonia mercurella	OY720055	Boyes et al., (2025)
		Eudonia angustea	OZ208102	Unpublished

	Scoparia pyralella	OZ248065	Unpublished
	Scoparia ancipitella	OZ337911	Unpublished
Crambinae	*Pseudargyria interruptella*	NC_029751	Gschloessl et al., (2020)
	Diatraea saccharalis	NC_013274	Park et al., (2019)
	Chilo suppressalis	NC_015612	Park et al., (2019)
	Chilo sacchariphagus	NC_029716	Park et al., (2019)
	Chilo auricilius	KJ174087	Yang et al., (2018)
Pyraustinae	*Pyrausta despicata*	NC_046050	Gschloessl et al., (2020)
	Loxostege sticticalis	NC_027174	Gschloessl et al., (2020)
	Loxostege turbidalis	MN646773	Wu et al., (2023)
	Loxostege aeruginalis	MN635734	Wu et al., (2023)
	Ostrinia palustralis	MH574940	Ju et al., (2019)
	Ostrinia penitalis	KM395814	Zhou et al., (2020)
	Ostrinia kasmirica	NC_059846	Yi et al., (2023)
	Ostrinia furnacalis	NC_056248	Muro et al., (2023)
	Ostrinia zealis	NC_048888	Gschloessl et al., (2020)
	Ostrinia scapulalis	NC_048887	Liu et al., (2021)
	Ostrinia nubilalis	NC_054270	Liu et al., (2021)
Spilomelinae	*Nomophila noctuella*	NC_025764	Ma et al., (2016)
	Pycnarmon lactiferalis	NC_033540	Gschloessl et al., (2020)
	Cnaphalocrocis medinalis	NC_015985	He et al., (2015)
	Cnaphalocrocis exigua	MN877384	Yi et al., (2023)
	Haritalodes derogata	KR233479	Yang et al., (2018)
	Pycnarmon pantherata	KX150459	Wu et al., (2023)

	Nagiella inferior	NC_040973	Gschloessl et al., (2020)
	Tyspanodes striata	NC_030510	Liu et al., (2021)
	Tyspanodes hypsalis	NC_025569	Ma et al., (2016)
	Maruca vitrata	NC_024099	He et al., (2015)
	Maruca testulalis	NC_024283	Park et al., (2019)
	Palpita nigropunctalis	KX150458	Wu et al., (2023)
	Palpita hypohomalia	NC_039632	Gschloessl et al., (2020)
	Cydalima perspectalis	NC_042150	Gschloessl et al., (2020)
	Conogethes punctiferalis	NC_021389	Liu et al., (2016)
	Conogethes punctiferalis	KX150457	Wu et al., (2023)
	Omiodes indicata	NC_039177	Bian et al., (2020)
	Glyphodes quadrimaculalis	NC_022699	Dong et al., (2016)
	Glyphodes pyloalis	NC_025933	Wu et al., (2016)

Table S2 Optimal partitioning schemes and substitution models for the PCG123 dataset (13 protein-coding genes) of 58 Pyraloidea species used in maximum likelihood (ML) phylogenetic analyses.

Optimal partition	Model	Subset partition
Partition1	GTR + F + R4	nad3, cox3, atp6
Partition2	TIM3 + F + G4	atp8
Partition3	GTR + F + I + G4	cox1
Partition4	TIM2 + F + I + R4	cox2
Partition5	GTR + F + R5	cytb
Partition6	GTR + F + R4	nad1

Partition7	TIM + F + I + R4	nad2, nad3
Partition8	GTR + F + R5	nad4, nad5, nad4L
Partition9	GTR + F + I + G4	nad6

Table S3 Optimal partitioning schemes and substitution models for PCG123 dataset (13 protein-coding genes) of 58 Pyraloidea species used in Bayesian inference (BI) phylogenetic analyses.

Optimal partition	Model	Subset partition
Partition1	GTR + F + I + G4	atp6
Partition2	GTR + F + G4	atp8
Partition3	GTR + F + I + G4	cox1
Partition4	GTR + F + I + G4	cox2
Partition5	GTR + F + I + G4	cox3, cytb
Partition6	GTR + F + I + G4	nad1
Partition7	GTR + F + I + G4	nad2, nad3
Partition8	GTR + F + I + G4	nad4L
Partition9	GTR + F + I + G4	nad4, nad5, nad6

Table S4 Optimal partitioning schemes and substitution models for PCG123 + rRNA dataset (13 protein-coding genes + 2 ribosomal RNAs) of 58 Pyraloidea species used in maximum likelihood (ML) phylogenetic analyses.

Optimal partition	Model	Subset partition
Partition1	GTR + I + G	cox1, nad3, cox3, atp6
Partition2	GTR + I + G	nad2, nad6, atp8
Partition3	GTR + I + G	cytb, cox2
Partition4	GTR + I + G	nad4, nad5, nad4L, nad1
Partition5	GTR + G	rrnS, rrnL

Table S5 Optimal partitioning schemes and substitution models for PCG123 + rRNA dataset (13

protein-coding genes + 2 ribosomal RNAs) of 58 Pyraloidea species used in Bayesian inference (BI) phylogenetic analyses.

Optimal partition	Model	Subset partition
Partition1	GTR + I + G	cox1, cox3, atp6, nad3
Partition2	GTR + I + G	nad2, nad6, atp8
Partition3	GTR + I + G	cytb, cox2
Partition4	GTR + I + G	nad5, nad4, nad4L, nad1
Partition5	GTR + G	rrnS, rrnL

Table S6 Geographic Distribution of *E. extimalis.*

Serial Number	Country	Sample points	Province	Longitude	Latitude	Altitude(m)
1	China (CN)	47	Beijing (BJ)	116.41	39.90	50
2			Beijing (BJ)	116.39	39.90	50
3			Guangdong (GD)	113.28	23.12	12
4			Guangdong (GD)	114.08	22.63	83
5			Hebei (HEB)	114.48	38.05	80
6			Hebei (HEB)	115.26	40.56	756
7			Hebei (HEB)	115.32	41.05	1541
8			Hebei (HEB)	115.37	41.20	1779
9			Hebei (HEB)	115.78	40.58	1501
10			Hebei (HEB)	117.60	42.51	1487
11			Heilongjiang (HLJ)	126.66	46.64	139
12			Heilongjiang (HLJ)	130.59	45.76	212
13			Inner Mongolia (IM)	122.34	43.93	168
14			Inner Mongolia (IM)	111.77	40.85	1075
15			Jiangsu (JS)	120.27	32.05	3
16			Jiangsu (JS)	119.46	34.36	2
17			Jilin (JL)	125.33	43.88	223
18			Jilin (JL)	126.55	41.80	1118
19			Liaoning (LN)	123.44	41.81	51
20			Liaoning (LN)	122.24	39.91	345
21			Ningxia (NX)	106.26	38.47	1111
22			Ningxia (NX)	105.79	37.59	1208
23			Qinghai (QH)	101.68	36.93	2512

24			Qinghai (QH)	101.57	36.50	2747
25			Qinghai (QH)	101.25	36.68	2733
26			Qinghai (QH)	101.78	36.62	2313
27			Qinghai (QH)	102.02	36.84	2646
28			Qinghai (QH)	102.10	36.49	2203
29			Qinghai (QH)	101.62	37.38	2925
30			Qinghai (QH)	100.75	35.59	3256
31			Shaanxi (SNX)	108.95	34.28	407
32			Shaanxi (SNX)	109.49	36.09	1177
33			Shandong (SD)	117.13	36.67	70
34			Shandong (SD)	118.89	35.46	125
35			Shanghai (SH)	121.48	31.23	12
36			Shanghai (SH)	121.31	30.75	5
37			Shanxi (SX)	112.57	37.87	797
38			Shanxi (SX)	111.50	36.65	986
39			Shanxi (SX)	111.51	37.79	1863
40			Sichuan (SC)	104.07	30.68	503
41			Sichuan (SC)	102.96	29.59	1613
42			Tibet (TB)	88.09	31.69	4575
43			Tibet (TB)	95.09	29.66	5567
44			Xinjiang (XJ)	87.63	43.80	878
45			Xinjiang (XJ)	87.63	41.88	1358
46			Yunnan (YN)	102.73	25.05	1901
47			Yunnan (YN)	100.23	23.47	1632
48	Åland Islands (AX)	1		20.99	60.42	1
49	Austria (AT)	6		14.32	47.55	771
50				13.65	48.00	481
51				14.34	46.63	480
52				16.18	47.85	326
53				16.80	47.98	183
54				16.81	47.78	117
55	Belarus (BY)	1		28.33	54.10	174
56	Croatia (HR)	1		16.33	46.33	172
57	Denmark (DK)	4		8.19	55.81	6
58				11.97	54.57	2
59				11.97	54.57	2
60				15.10	55.15	41

61	Estonia (EE)	3	27.04	58.28	45
62			27.29	58.01	60
63			27.31	57.79	141
64	Finland (FI)	32	28.90	61.43	119
65			28.90	61.43	119
66			22.56	60.35	13
67			22.56	60.35	13
68			23.48	62.79	76
69			23.52	61.56	158
70			24.00	60.29	61
71			24.01	60.30	61
72			24.50	63.03	182
73			24.83	60.60	103
74			24.83	61.59	135
75			25.21	60.70	79
76			25.21	60.70	79
77			26.12	61.74	122
78			26.17	60.37	10
79			26.90	61.44	104
80			26.91	60.64	30
81			27.04	61.76	122
82			27.63	62.92	101
83			27.63	60.54	29
84			27.69	62.97	91
85			27.69	62.97	91
86			28.20	61.08	82
87			28.21	61.07	84
88			29.41	61.46	77
89			29.42	62.50	70
90			29.64	62.32	82
91			29.64	62.32	82
92			29.87	62.42	74
93			29.96	62.04	80
94			30.61	62.21	119
95			30.92	62.67	146
96	France (FR)	32	-0.84	47.24	99
97			2.93	44.81	1004
98			-3.60	48.80	0
99			-2.21	46.97	0
100			-2.19	48.61	9

101				-2.11	48.64	21
102				-2.10	48.63	21
103				-1.80	46.55	7
104				-1.75	47.20	17
105				-1.07	46.38	1
106				-0.95	47.38	21
107				-0.53	47.49	39
108				0.70	44.76	129
109				0.70	44.76	129
110				1.00	49.32	92
111				1.00	49.35	90
112				1.53	50.22	-9999
113				2.00	44.78	459
114				3.50	43.67	125
115				3.87	43.53	4
116				4.00	43.59	3
117				4.01	43.72	74
118				4.40	43.49	0
119				4.43	43.68	20
120				4.56	43.83	48
121				4.58	43.81	33
122				4.75	43.63	19
123				4.76	43.54	-1
124				4.77	43.67	20
125				5.33	43.71	316
126				5.41	44.40	817
127				7.67	48.58	142
128	Germany (DE)	9		13.99	51.82	67
129				7.68	51.93	55
130				8.22	50.02	99
131				9.88	52.45	61
132				9.88	52.45	61
133				10.58	53.74	52
134				11.15	52.69	53
135				13.65	52.48	46
136				14.21	51.42	131
137	Ireland (IE)	2		-7.85	52.95	114
138				-8.02	54.13	67
139	Italy	2		12.35	44.82	-2
140				13.45	42.34	693

141	Japan (JP)	9	139.92	40.04	6
142			140.99	43.24	125
143			141.09	40.20	347
144			141.10	40.22	391
145			141.13	40.17	271
146			141.19	39.70	227
147			141.48	39.60	721
148			141.84	40.05	98
149			144.60	43.30	43
150	Lithuania (LT)	2	21.07	56.00	1
151			24.49	54.77	129
152	Netherlands (NL)	4	3.60	51.45	1
153			3.60	51.50	0
154			6.25	52.10	11
155			36.09	51.57	235
156	Norway (NO)	1	10.58	60.02	236
162	Poland (PL）	3	19.56	53.60	105
163			19.59	53.60	101
164			20.31	52.96	130
165	Russia (RU）	17	38.37	57.01	138
166			37.12	58.76	113
167			27.87	57.73	81
168			29.36	56.34	161
169			43.58	55.68	149
170			43.60	54.89	187
171			59.97	59.80	216
172			60.58	56.83	265
173			82.92	55.06	159
174			82.99	55.02	165
175			91.36	53.67	248
176			91.36	53.67	248
177			131.25	42.80	16
178			132.37	43.20	33
179			143.75	43.87	237
180			143.84	43.97	213
181			143.91	43.82	83
182	Slovenia (SI）	2	13.95	45.71	418
183			16.02	46.76	283
184	South Korea (KR）	9	126.55	34.51	72

185			126.84	37.84	85
186			127.19	34.73	116
187			127.44	36.76	120
188			127.56	37.14	125
189			127.69	37.18	106
190			128.15	37.46	555
191			128.19	35.19	181
192			128.19	35.32	177
193	Spain (ES）	2	-0.75	37.80	-9999
194			0.06	42.07	433
195	Sweden (SE）	21	13.09	55.97	85
196			13.10	55.88	47
197			11.13	58.87	16
198			14.23	55.43	15
199			14.32	55.95	10
200			15.10	56.50	155
201			15.54	59.56	81
202			15.54	56.20	11
203			16.19	56.55	12
204			16.44	57.05	8
205			16.44	60.44	120
206			16.64	56.62	10
207			16.64	56.62	10
208			17.29	60.73	20
209			17.29	60.44	34
210			17.70	59.96	45
211			17.71	59.96	43
212			18.21	57.02	4
213			18.21	57.60	39
214			18.90	59.91	19
215			18.90	59.34	13
216	Turkey (TR）	3	42.13	38.56	1742
217			28.84	40.97	18
218			127.56	37.05	147
219	Ukraine (UA）	1	36.35	50.03	149
220	United Kingdom (GB）	14	-2.57	56.96	191
221			-0.55	51.11	50
222			-5.23	49.98	64

223			-3.84	50.23	60
224			-3.09	50.89	223
225			-2.81	54.17	27
226			-0.46	52.36	65
227			-0.13	51.24	85
228			-0.09	51.37	76
229			-0.02	52.36	18
230			-0.02	52.45	0
231			0.48	52.36	6
232			0.48	52.44	0
233			1.55	52.09	1
234	United States (US）	14	-118.48	36.20	1987
235			-118.06	36.61	1143
236			-117.69	34.39	1937
237			-117.63	34.36	2108
238			-110.87	31.71	2007
239			-109.74	32.88	937
240			-109.25	31.91	2488
241			-108.54	32.81	1605
242			-108.53	45.08	1585
243			-108.47	32.69	1812
244			-108.24	32.90	2155
245			-106.33	35.19	2136
246			-104.84	38.71	2051
247			-103.89	30.59	1537

Table S7 List of mitochondrial genomes of Crambidae species for comparison with *E. extimalis*.

Species	Family	Size (bp)	Accession No.	Reference
E. junctalis	Crambidae	15,438	KP347963	Song and You. 2015
Dausara latiterminalis	Crambidae	15,147	MW732137	Qi et al. 2021
Conogethes punctiferalis	Crambidae	15,332	MT670378	Jeong et al. 2021
Chilo auricilius	Crambidae	15367	KJ174087	Cao and Du. 2014
Prophantis octoguttalis	Crambidae	15,197	OP559507	Tang and Du. 2023
Scirpophaga incertulas	Crambidae	15,223	KC493629	Cao et al. 2014
Paracymoriza prodigalis	Crambidae	15,326	JX144892	Ye et al. 2013
Eumorphobotys obscuralis	Crambidae	15,348	OR459845	Li and Zhang, 2023

Pyrausta despicata	Crambidae	15,389	MN956508	Wu et al. 2022
Nyctegretis seminigra	Crambidae	15,198	MN646773	Yang et al. 2025
Elophila interruptalis	Crambidae	15.351	KC894961	Park et al. 2014

Table S8 Composition and skewness in the Crambidae mitochondrial genomes.

Species	A%	T%	C%	G%	A+T%	AT skewness	GC skewness
E. junctalis	39.93	41.15	11.05	7.87	81.08	-0.015	-0.168
Dausara latiterminalis	40.10	40.40	11.70	7.78	80.60	-0.003	-0.201
Conogethes punctiferalis	40.69	41.76	10.32	7.23	82.45	-0.013	-0.176
Chilo auricilius	40.67	41.36	10.75	7.22	82.03	-0.008	-0.196
Prophantis octoguttalis	41.0	40.5	11.0	7.5	81.5	0.006	-0.189
Scirpophaga incertulas	39.71	37.34	15.19	7.76	77.05	0.031	-0.324
Paracymoriza prodigalis	40.84	40.70	10.92	7.54	81.53	0.002	-0.183
Eumorphobotys obscuralis	40.99	40.06	11.47	7.49	81.05	0.011	-0.21
Pyrausta despicata	40.84	40.13	11.45	7.58	80.97	0.009	-0.203
Nyctegretis seminigra	37.58	41.62	12.70	8.10	79.2	-0.051	-0.221
Elophila interruptalis	39.73	40.59	12.09	7.59	80.32	-0.011	-0.229
PCGs							
E. junctalis	34.33	46.23	9.07	10.38	80.56	-0.1478	0.0675
Dausara latiterminalis	34.22	44.98	10.10	10.71	79.09	-0.136	0.029
Conogethes punctiferalis	33.24	45.91	10.05	10.98	78.9	-0.061	0.019
Chilo auricilius	40.14	40.48	11.41	7.97	80.61	-0.004	-0.178
Prophantis octoguttalis	40.5	39.8	11.6	8.2	80.3	0.01	-0.173
Scirpophaga incertulas	39.17	35.70	16.56	8.57	74.87	0.046	-0.318
Paracymoriza prodigalis	40.34	39.78	11.59	8.29	80.12	0.007	-0.166
Eumorphobotys obscuralis	33.74	45.67	9.91	10.68	79.41	-0.150	0.037
Pyrausta despicata	33.95	45.55	9.96	10.54	79.50	-0.1459	0.0283
Nyctegretis seminigra	36.78	40.82	10.2	12.20	77.6	-0.152	-0.089

Elophila interruptalis	39.28	39.33	12.95	8.44	78.61	-0.001	-0.211
tRNAs							
E. junctalis	41.98	39.62	7.75	10.65	81.60	0.029	0.158
Dausara latiterminalis	40.53	40.53	8.27	10.66	81.07	0.000	0.126
Conogethes punctiferalis	36.81	41.63	10.58	10.98	78.44	-0.061	0.019
Chilo auricilius	42.32	40.78	9.59	7.31	83.10	0.011	-0.134
Prophantis octoguttalis	41.4	40.7	9.9	7.9	82.2	0.009	-0.112
Scirpophaga incertulas	40.97	39.66	11.38	8.00	80.62	0.016	-0.174
Paracymoriza prodigalis	41.82	40.04	10.13	8.01	81.86	0.022	-0.117
Eumorphobotys obscuralis	36.44	41.21	10.47	11.88	77.65	-0.061	0.063
Pyrausta despicata	40.95	41.15	7.23	10.65	82.09	-0.002	0.191
Nyctegretis seminigra	40.64	39.76	8.00	11.60	80.4	0.011	0.184
Elophila interruptalis	41.07	41.00	10.15	7.78	82.07	0.001	-0.132
rRNAs							
E. junctalis	44.42	40.56	4.99	10.03	84.97	0.0455	0.3354
Dausara latiterminalis	43.64	41.37	4.94	10.00	85.01	0.0267	0.3387
Conogethes punctiferalis	41.87	42.20	4.94	10.99	84.07	-0.004	0.380
Chilo auricilius	40.89	44.59	4.82	9.70	85.48	-0.043	-0.33-
Prophantis octoguttalis	42.5	42.4	9.9	5.0	84.9	0.001	0.3386
Scirpophaga incertulas	40.92	41.88	4.85	12.34	82.80	-0.012	-0.436
Paracymoriza prodigalis	41.43	44.24	4.75	9.59	85.67	-0.033	-0.338
Eumorphobotys obscuralis	42.26	42.45	4.99	10.31	84.71	-0.002	0.348
Pyrausta despicata	42.28	42.06	4.93	10.69	84.34	0.003	0.369
Nyctegretis seminigra	45.25	39.25	4.90	10.60	84.5	0.071	0.368
Elophila interruptalis	40.64	43.99	10.68	4.69	84.63	-0.040	-0.390
A+T-rich region							
E. junctalis	43.87	50.43	3.42	2.28	94.30	-0.0695	-0.200
Dausara latiterminalis	43.58	51.64	3.28	1.49	95.22	-0.085	-0.375
Conogethes punctiferalis	27.11	63.56	2.33	0.58	90.67	-0.402	-0.601

Chilo auricilius	46.29	47.77	4.75	1.19	94.07	-0.016	-0.599
Prophantis octoguttalis	46.2	49.8	3.1	0.9	96.0	-0.038	-0.55
Scirpophaga incertulas	42.43	50.37	5.21	1.99	92.80	-0.086	-0.447
Paracymoriza prodigalis	45.48	49.85	3.50	1.17	95.34	-0.046	-0.499
Eumorphobotys obscuralis	42.71	53.56	2.71	1.02	96.27	-0.113	-0.455
Pyrausta despicata	42.54	50.99	5.53	1.13	93.52	-0.0903	-0.6607
Nyctegretis seminigra	43.11	49.99	4.40	2.50	93.1	-0.074	-0.275
Elophila interruptalis	43.66	49.85	5.31	1.18	93.51	-0.066	-0.636

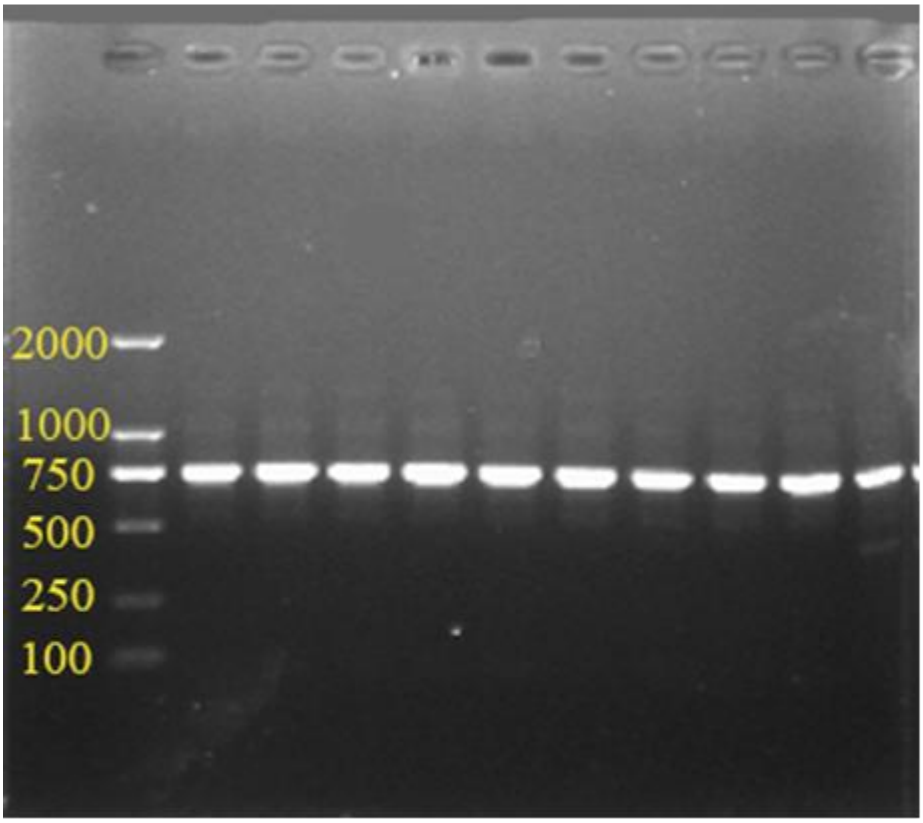

Figure S1. Polymerase chain reaction (PCR) analysis to confirm the larvae of *E. extimalis* by targeting the *COI* (cytochrome c oxidase subunit I) gene.
